# Teaching Life-Saving Manoeuvres in Primary School

**DOI:** 10.1155/2016/2647235

**Published:** 2016-11-13

**Authors:** Sara Calicchia, Giovanna Cangiano, Silvia Capanna, Mariangela De Rosa, Bruno Papaleo

**Affiliations:** INAIL, Italian Workers' Compensation Authority, Department of Occupational and Environmental Medicine, Epidemiology and Hygiene, Via Fontana Candida 1, Monte Porzio Catone, 00078 Rome, Italy

## Abstract

*Introduction.* In the event of sudden cardiac arrest (SCA) early intervention provided by a layperson can be life-saving. Teaching first aid in primary school may increase the lifelong ability and motivation of young people to take action in an emergency.* Objective.* The aim of this article is to report a training experience on BLSD (Basic Life Support and Defibrillation) designed for a group of pupils in an Italian primary school, with assessment of its effectiveness at a distance.* Methods.* The assessment was carried out using a multiple choice questionnaire on a sample of 130 pupils aged 11-12, 62 trained in BLSD and 68 as a control group. The trained group also performed an emergency simulation to assess their learning of practical skills.* Results.* Using the *t* test, significant differences emerged in the questionnaire scores between the case-control group. The results of the skill test were positive, even for the most difficult manoeuvres such as opening airways, assessing breathing, or using an AED (Automated External Defibrillator).* Conclusion.* Although there are still some open questions regarding the ability to retain these skills in the medium/long term, the study shows that life-saving manoeuvres can be effectively taught to primary school pupils.

## 1. Introduction

In the event of sudden cardiac arrest (SCA) early intervention (within 3–5 minutes) with CPR (Cardiopulmonary Resuscitation) and defibrillation increases survival rates [1–3]. That is why it is important for all citizens to be able to recognise a cardiac emergency and administer first aid while the advanced life support arrives. Many studies have shown that what actually prevents the ordinary citizen from intervening in the event of an emergency is the fear of doing something wrong, whereas bystanders who are trained in manoeuvres are more likely to take action [4].

The question that arises then is how to spread knowledge of these manoeuvres to a large proportion of the population, in a percentage that would increase the rate of intervention. According to a study provided by the Red Cross on First Aid in Europe, the countries with a higher percentage of citizens that are able to respond to an emergency (Norway, Germany, Austria, and Iceland) have laws that make first aid training compulsory either at school, in the workplace, or when applying for a driving license [5, 6]. That is why, in many countries where teaching is not yet compulsory, the scientific community and resuscitation and rescue associations are working to raise awareness within institutions of the importance of including teaching First Aid manoeuvres in school curricula [7, 8]. In Italy only recently the teaching of first aid at school has been planned [9]. Sensitivity to these issues has grown in recent years, driven by a series of legislative initiatives that have focused attention on the use of AED (Automated External Defibrillator), especially in high-traffic public environments, such as schools, or where there is high risk, and by several projects conducted by associations and individual educational institutes.

When it comes to training very young individuals the greatest doubt is about their ability to learn how to provide assistance like an adult, especially when thinking in terms of the cost/benefit ratio. Providing data on the effectiveness of training, including the purposes of adapting educational programmes for a younger audience, proves to be an essential step in facilitating and guiding educational institutions and the relevant ministries in introducing specific training programmes. The aim of this work is to report a training experience on BLSD (Basic Life Support and Defibrillation) with assessment of the effectiveness at a distance, carried out at a primary school in the Province of Rome.

## 2. Materials and Methods

### 2.1. Training

The experience was conducted by instructors from the IRC training network (Italian Resuscitation Council) for the Community and was included in the PAD (Public Access to Defibrillation) project, called “The heart of Monteporzio” and run by the Municipality of Monte Porzio Catone (Rome), to underline the concept of the community taking responsibility in the event of sudden cardiac death and to communicate an idea of a “community of hearts,” both in a practical and a symbolic way. In just a few months 14 AEDs have been distributed across the local area and more than 100 people have received training, including members of the Police and Civil Protection, teachers and school staff, coaches, and ordinary citizens. Two AEDs have been deployed within the school, one for each location.

The challenge was to structure a successful educational path, usually structured for an adult target, which was adequate to not only transfer practical skills, but also to involve young people on a motivational level. Fifth grade junior school and third grade middle school pupils, for a total of 141 children from 9 to 12 years old, were chosen as the primary target for training. The course was designed with preference to an active learning method, based on simulations and exercises in groups [10]. With the aim of raising awareness within the children's living environment, to emphasise the importance and the social aspect of knowing what to do in case of emergency, teachers and parents were also involved in the process.

The activities, carried out over a period of 3 months and described below, have been defined on the three areas of expertise (knowledge, know-how, and know how to be) and the respective learning objectives [11] (Table 1).

#### 2.1.1. Preliminary Activities in the Classroom

Teachers were involved right from the planning stages, with a series of brainstorming meetings which allowed us to

- transfer and verify the feasibility of learning objectives,
- raise awareness and inform teachers on the topic of first aid,
- share teaching materials and worksheets, also used in a similar experience within the same training network [12],
- plan an educational path integrated with the school curriculum.

During this phase, concepts regarding the cardiovascular apparatus, cardiac arrest, and emergency situations were transferred. Furthermore, a series of corresponding activities was organised to give wide range to the youngsters' creativity and to their community spirit:

- “Design a logo for the project: The Heart of Monteporzio,” a contest that allowed primary school children to imagine the graphic line of the PAD project.
- “You don't need to be a superhero to save a life,” a contest sponsored by the Ministry for Education whereby the third grade middle school pupils produced spot advertising and the fifth grade junior school children drew animated cartoons.
- Creation of a teaching video on First Aid: with their cameras at the ready, the instructors played the role of directors and the children actors, to simulate an emergency scene.

#### 2.1.2. Workshops with BLSD Instructors

The workshops were held by instructors who are qualified in compliance with ERC (European Resuscitation Council) 2010 Guidelines. A total of four meetings were held, each of which was about two hours:

- *First Workshop: What Do We Know?* The children's experiences of first aid were collected via a brainstorming session in the classroom as they began to think about case studies, discussing them in small groups with the support of facilitators and instructors. In this way it was possible to “fix some points” about the state of consciousness/unconsciousness, the call to the emergency number, and the emotional state of the moment.
- *Second Workshop: Now Let's Learn What to Do.* After a brief theoretical introduction, practical training was carried out on a manikin, in small groups. The whole sequence, consisting in assessment of environmental safety, assessment of consciousness, alerting emergency number, assessment of breathing, chest compressions (without rescue breathings), and use of the AED, was taught only to third year middle school students. The main doubts concerned the transfer of skills to fifth grade junior school children in relation to chest compressions and the use of the AED. Since some studies report a reduced ability to exercise effective chest compressions and providing rescue breaths in this age group [13, 14], we opted for teaching the use of the AED, but not chest compressions and ventilation.
- *Third Workshop: What Have We Learnt?* In collaboration with the municipal administration, the police, and local associations, a sort of public celebration was organised in the school gym, which included
  - the awarding of the best logo which has also become the official logo of the PAD project,
  - the projection of the video on First Aid filmed in the school,
  - the delivery by the Mayor of “heart-saving citizen” certificates and t-shirts with the project logo.

In this way, the children were able to incorporate their experience into a social and civic dimension, strengthening the awareness of their own skills.

*Fourth Workshop: Let's Tell Our Parents about Our Experience.* During the final meeting, aimed at raising awareness within the family environment and sharing the path with their parents, the pupils were given the floor to talk about their experience using materials they produced themselves: slides, videos, and so forth.

### 2.2. Assessment

A year later the effectiveness of the training experience was assessed using a multiple choice questionnaire on a sample of 62 pupils aged 11-12 (37 males, 25 females). Only the children from the fifth year juniors were assessed, due to the difficulty in tracing the third year middle school youngsters who had now moved on to upper schools. The questionnaire was subsequently administered to a control group of 68 pupils of the same age (37 males, 31 females), from a different school, who had never taken part in first aid courses. The questions investigated different aspects of knowledge and know-how and also considered willingness to personally take action in the event of an emergency. The case group was also given a skill test to assess the learning of practical skills and reaction times, expertise that is difficult to assess with a questionnaire.

## 3. Results

### 3.1. Questionnaires

Analysis was carried out using the SPSS® 21.0 software (IBM® Corporation, Armonk, NY, USA). For each correct answer a score of 1 was given, up to a total of 15 points. None of the children obtained a full score. The highest score was 13.5 (90% of the answers correct) obtained by 4 children belonging to the “case” group only. Using the *t* test, significant differences emerged in the scores obtained in the questionnaires between the case-control group (*p* < 0.005), whereas there were none between genders.

When looking more closely at some of the questions, where there was a greater difference in scores, it emerged that the children who received training showed greater ability in handling and dealing with an emergency situation, even after a year. Faced with concrete cases, for example, they are more likely to assess the environment, ensuring that they are safe from danger too. As far as calling the emergency number is concerned, this can be done by both samples; however, the case group is better prepared to provide information in the right way.

### 3.2. Skill Test

**Figure 1:**
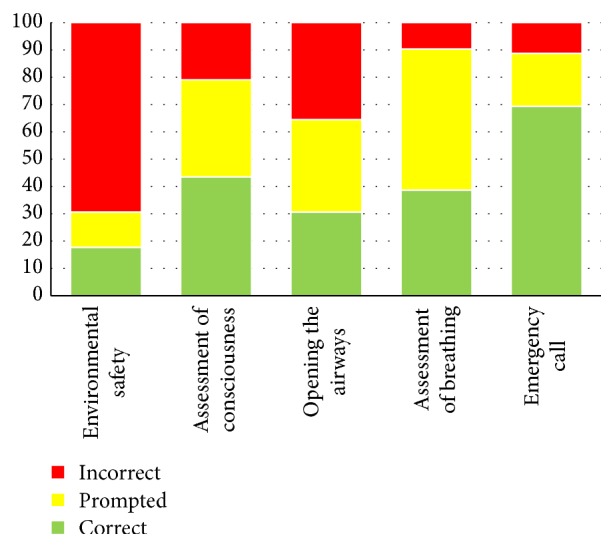
Percentage of manoeuvres correct, prompted, and incorrect.

The skill tests were carried out by qualified instructors who had also taken note of the critical issues most frequently encountered during the execution of the manoeuvres. For each manoeuvre performed it was possible to assign three different colours: green, when it was done properly and without suggestion; yellow, in the case of minor errors or in the case of manoeuvres remembered after receiving advice; red, in the case of severely incorrect or forgotten manoeuvres.  Figure 1 shows the percentage of manoeuvres correct, prompted, and incorrect.

As seen in other studies [15], the stress factor caused by the presence of the instructors should be taken into consideration, as well as the time elapsed since the course was held. Despite this, the overall results appear to be positive, even for the most difficult manoeuvres from a technical point of view, such as opening the airways and assessing breathing.

**Figure 2:**
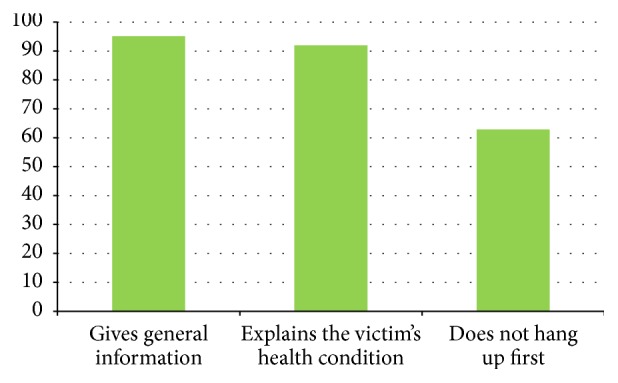
Call to the emergency number (percentage).

The evaluation of environmental safety, though in the questionnaires there was the main difference with the control group, is often overlooked, probably because the children felt they are in a safe environment (the school gym). Data confirm the critical issues noted by the instructors and concern, above all, hyperextension of the head and assessment of breathing, which are often only remembered when prompted by the instructor. Advanced life support providers are alerted spontaneously by most of the students (69.35%) with just a very small percentage needing advice (19.35%). Furthermore, more than 60% of the children, when left to freely express the content of the call to the emergency number, remembered the key information to be provided (Figure 2).

**Figure 3:**
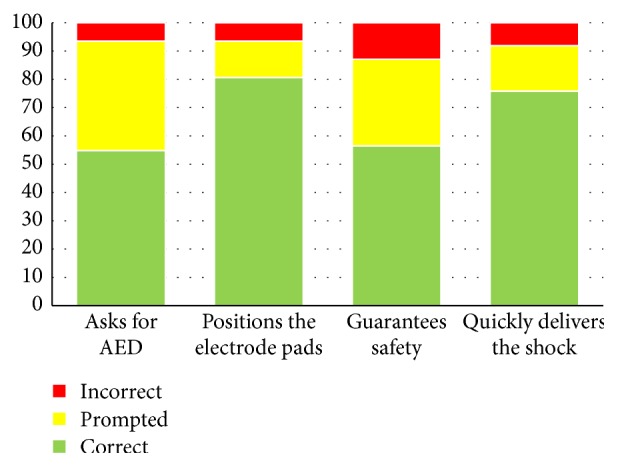
AED use results (percentage).

As far as using the AED is concerned, no particular problems have been reported. The majority of students are able to position the electrode pads correctly, deliver the shock safely, and handle the situation (Figure 3).

## 4. Discussion

The study shows that life-saving manoeuvres can be effectively taught to primary school pupils. In addition, the use of the AED proved to be easy to learn, even by the smallest children, as they quickly and easily internalised what it was for, where and how to position the electrode pads, and, above all, the need to ensure safety.

In this regard, and in view of the limited data available in literature, there was some uncertainty within the group of instructors on whether to teach the use of this device, which is generally used by an adult audience. The results were surprisingly positive: thanks to the greater familiarity of youngsters with technology, the safe learning of the AED seemed to be “child's play.”

Although there are still some open questions regarding the ability to retain these skills in the medium/long term, our study confirms other data reported in literature that there is no “ideal” age when First Aid training is more effective [13, 15, 16]. Indeed, researches on memorising psychomotor skills suggest that early training contributes to maintaining a high level of skills over time [17] and children who have received training are more ready to intervene in case of emergency than their peers [15]. Apart from practical skills, which require constant retraining to be maintained over time, BLSD courses can change the attitudes and behaviour of youngsters at a time of life when they easily absorb new information [18]. The conclusions of a study conducted in Norway observing the behaviour of trained children (4-5 years) confirm that beginning education to first aid at an early age leads to include it in the activities and in the habits of everyday life and contributes to keep empathy towards others active [16]. Another study, conducted in a school in Barcelona, concludes that school offers the best setting to study these manoeuvres and such training increases the self-esteem of children and it could potentially contribute to saving lives [19].

Involving students in programmes to teach life-saving manoeuvres can respond, even in the long term, to the need to increase the percentage of the population that is able to respond in case of emergency, as school offers privileged access to a large proportion of the community, including members of families [17]. The school setting, like other places for socialisation, such as the workplace, is able to provide to a wide range of potential rescuers and future adults the opportunity of learning and absorbing the concepts of First Aid over time, because it facilitates frequent retraining [20]. Even in the short term it is important that children at least know how to alert emergency system correctly, since many cardiac arrests occur at home, often in the presence of relatives and friends [18].

In Italy we are still a long way from including these skills in school curricula and even amongst teachers learning emergency manoeuvres is left to the will of the individual and the sensitivity of the local community. The involvement of teachers could, however, be a key factor to spread the culture of emergency and to facilitate frequent retraining. In fact, many studies have shown that teachers, if properly trained, are perfectly able to teach their students the main manoeuvres [13]. In a study conducted in Ireland, for example, a model of “chain” teaching BLS was successfully tested where the teachers who trained the children were in turn trained by undergraduates in medicine [21, 22]. There is also a whole range of tools (such as self-training kits or animated videos) that can support teachers in their task [23].

## 5. Conclusions

In Italy, legislation on the use of AEDs and the widespread use of PAD projects, also in schools, are giving a great boost to the spread of the emergency culture. Our experience confirms that teaching kids is possible, effective, and fun. The critical issue is raising awareness and encouraging the participation of institutional partners (school administrators, teachers, local administrators, and parents) who show resistance and mistrust that are difficult to overcome. Including the teaching of key BLSD's manoeuvres in the school curriculum could respond to the aim of improving safety culture in school environments, raising awareness among adults and, at the same time, transferring this culture to younger generations, leading, in the long term, to structural change.

## Figures and Tables

**Table 1:** Areas of expertise and learning objectives.

Areas of expertise	Learning objectives
Knowledge	Theoretical knowledge regarding the following:
	(i) Cardiovascular system, respiratory system, and nervous system
	(ii) Cardiac arrest: causes and therapy
	(iii) Importance of activating the chain of survival

Know-how	Practical skills regarding the correct sequence of action:
	(i) Assessment of environmental safety
	(ii) Assessment of consciousness
	(iii) Alerting emergency number
	(iv) Assessment of breathing
	(v) Cardiac massage (only for third year middle school students)
	(vi) Use of AED

Know how to be	Skills related to the proper way to deal with an emergency without being panic-stricken:
	(i) Raising awareness of the importance of acting quickly in an emergency and activating PAD projects

## References

[1] Nolan J. P., Soar J., Zideman D. A. (2010). European resuscitation council guidelines for resuscitation 2010 section 1. Executive summary. *Resuscitation*.

[2] Caffrey S. L., Willoughby P. J., Pepe P. E., Becker L. B. (2002). Public use of automated external defibrillators. *The New England Journal of Medicine*.

[3] Capucci A., Aschieri D., Piepoli M. F., Bardy G. H., Iconomu E., Arvedi M. (2002). Tripling survival from sudden cardiac arrest via early defibrillation without traditional education in cardiopulmonary resuscitation. *Circulation*.

[4] Van de Velde S., Heselmans A., Roex A., Vandekerckhove P., Ramaekers D., Aertgeerts B. (2009). Effectiveness of nonresuscitative first aid training in laypersons: a systematic review. *Annals of Emergency Medicine*.

[5] International Federation of Red Cross and Red Crescent Societies. First aid for a safer future, Focus on Europe 2009, https://www.ifrc.org/PageFiles/53459/First%20aid%20for%20a%20safer%20future%20Focus%20on%20Europe%20%20Advocacy%20report%202009.pdf?epslanguage=en

[6] British Red Cross http://www.scribd.com/doc/59794984/Right-Place-Right-Time.

[7] Campbell S. (2012). Supporting mandatory first aid training in primary schools. *Nursing Standard*.

[8] Uray T., Lunzer A., Ochsenhofer A. (2003). Feasibility of life-supporting first-aid (LSFA) training as a mandatory subject in primary schools. *Resuscitation*.

[9] Legge 13 Luglio 2015, n. 107—art. 1, comma 10, http://www.gazzettaufficiale.it/eli/id/2015/07/15/15G00122/sg

[10] Lubrano R., Romero S., Scoppi P. (2005). How to become an under 11 rescuer: a practical method to teach first aid to primary schoolchildren. *Resuscitation*.

[11] Battistelli A., Majer V., Odoardi C. (1997). *Saper Fare, Essere*.

[12] Brianza per il Cuore (2005). *Apprendere a Portare Soccorso al Cuore*.

[13] Fleischhackl R., Nuernberger A., Sterz F. (2009). School children sufficiently apply life supporting first aid: a prospective investigation. *Critical Care*.

[14] Jones I., Whitfield R., Colquhoun M., Chamberlain D., Vetter N., Newcombe R. (2007). At what age can schoolchildren provide effective chest compressions? An observational study from the Heartstart UK schools training programme. *British Medical Journal*.

[15] Bollig G., Myklebust A. G., Østringen K. (2011). Effects of first aid training in the kindergarten—a pilot study. *Scandinavian Journal of Trauma, Resuscitation and Emergency Medicine*.

[16] Bollig G., Wahl H. A., Svendsen M. V. (2009). Primary school children are able to perform basic life-saving first aid measures. *Resuscitation*.

[17] Cave D. M., Aufderheide T. P., Beeson J. (2011). Importance and implementation of training in cardiopulmonary resuscitation and automated external defibrillation in schools: A Science Advisory from the American Heart Association. *Circulation*.

[18] Hazinski M. F., Markenson D., Neish S. (2004). Response to cardiac arrest and selected life-threatening medical emergencies: the medical emergency response plan for schools a statement for healthcare providers, policymakers, school administrators, and community leaders. *Circulation*.

[19] Maconochie I., Bingham B., Simpson S. (2007). Teaching children basic life support skills. *British Medical Journal*.

[20] Lester C. A., Weston C. F. M., Donnelly P. D., Assar D., Morgan M. J. (1994). The need for wider dissemination of CPR skills: are schools the answer?. *Resuscitation*.

[21] Connolly M., Toner P., Connolly D., McCluskey D. R. (2007). The ‘ABC for life’ programme-teaching basic life support in schools. *Resuscitation*.

[22] Toner P., Connolly M., Laverty L., McGrath P., Connolly D., McCluskey D. R. (2007). Teaching basic life support to school children using medical students and teachers in a ‘peer-training’ model—results of the ‘ABC for life’ programme. *Resuscitation*.

[23] Isbye D. L., Rasmussen L. S., Ringsted C., Lippert F. K. (2007). Disseminating cardiopulmonary resuscitation training by distributing 35,000 personal manikins among school children. *Circulation*.

